# Oil of Naphtha in Diarrhea

**Published:** 1849-06

**Authors:** 


*Oil of Naphtha in Diarrhea.*—The *Gazette des Hopitaux*, published in September last, contains a letter which speaks in very favorable terms of the efficacy of the oil of naphtha in the treatment of cholera and choleric diarrhea, as they prevailed in the Caucasian division of the Russian army during the summer of 1848.

Dr. Lavirotte, of Lyons, who has since used the oil in diarrhea, publishes the results of his experience in a late number of the same journal.

Of ten cases in which he administered this remedy, the disease notably abated or entirely disappeared in eight, in the course of twenty-four or forty-eight hours; whilst in only two was there no perceptible amelioration.

Cases I and II. Two phthisics, who presented caverns at the summit of both lungs, were tormented by sweats and an abundant diarrhea. Six drops of the oil of naphtha in an infusion of peppermint was administered to each. The diarrhea disappeared in twenty-four hours. At the end of two days the use of the remedy was suspended. The oil of naphtha and of petroleum have been much vaunted in phthisis pulmonalis. We must, however, in truth avow, that the two patients just referred to derived no other advantage from the use of the former than the arrest of the diarrhea. The progress of their disease was not retarded; they died, the one in eight, the other in ten days after the administration of the oil of naphtha, without the diarrhea reappearing.

Cases III, IV, V, VI, VII, and VIII, relate to patients laboring under typhoid fever complicated, with excessively copious diarrhea, in all of whom the discharges were very promptly arrested by the use of oil of naphtha in doses varying from six to twelve drops.

The disease proved intractable to the oil of naphtha and many other remedies in case No. 9.

The second instance in which the oil was inefficacious, was that of a man laboring under intermittent fever, complicated by œdema of the lower extremities and diarrhea. Opiates and astringents had failed, and after a fair trial of the oil, it was relinquished. The disease finally yielded, as it so often does in like cases, to tonics and a restorative regimen.

Thus, in eight cases out of the ten, in which the remedy was administered, it proved successful—the diarrhea being suppressed by it alone.

Dr. Lavirotte closes his letter by the following remarks:

"Does this medicine act like opium and the other calmants, as alum and colombo, and the other astringents, as nitrate of silver, by substitution, or rather, has it a special action which modifies the intestinal secretion?

"The oil of naphtha is too easily borne to allow of its being compared to nitrate of silver; its antispasmodic action might lead us to suppose it possessed of virtues analogous to those of calmants; but if it is recollected that naphtha is distinguished for its nauseous, bitter, and sharp savor, we will be induced rather to attribute to it an astringent action."

Dr. L. deems himself unable to answer whether or not it exerts a special action upon the intestinal mucous membrane. D. W. Y.

